# Sources of Pre-Analytical Variations in Yield of DNA Extracted from Blood Samples: Analysis of 50,000 DNA Samples in EPIC

**DOI:** 10.1371/journal.pone.0039821

**Published:** 2012-07-13

**Authors:** Elodie Caboux, Christophe Lallemand, Gilles Ferro, Bertrand Hémon, Maimuna Mendy, Carine Biessy, Matt Sims, Nick Wareham, Abigail Britten, Anne Boland, Amy Hutchinson, Afshan Siddiq, Paolo Vineis, Elio Riboli, Isabelle Romieu, Sabina Rinaldi, Marc J. Gunter, Petra H. M. Peeters, Yvonne T. van der Schouw, Ruth Travis, H. Bas Bueno-de-Mesquita, Federico Canzian, Maria-José Sánchez, Guri Skeie, Karina Standahl Olsen, Eiliv Lund, Roberto Bilbao, Núria Sala, Aurelio Barricarte, Domenico Palli, Carmen Navarro, Salvatore Panico, Maria Luisa Redondo, Silvia Polidoro, Laure Dossus, Marie Christine Boutron-Ruault, Françoise Clavel-Chapelon, Antonia Trichopoulou, Dimitrios Trichopoulos, Pagona Lagiou, Heiner Boeing, Eva Fisher, Rosario Tumino, Claudia Agnoli, Pierre Hainaut

**Affiliations:** 1 International Agency for Research on Cancer, Lyon, France; 2 Medical Research Council (MRC) Epidemiology Unit, Addenbrooke’s Hospital, Cambridge, United Kingdom; 3 Centre National de Génotypage, Institut Génomique, Commissariat à l’énergie Atomique, Evry, France; 4 Division of Cancer Epidemiology and Genetics, National Cancer Institute, National Institutes of Health, Department Health and Human Services, Bethesda, Maryland, United States of America; 5 Department of Epidemiology and Biostatistics, School of Public Health, Imperial College, London, United Kingdom; 6 Julius Center for Health Sciences and Primary Care, University Medical Center Utrecht, Utrecht, The Netherlands; 7 Cancer Epidemiology Unit, Nuffield Department of Clinical Medicine, University of Oxford, Oxford, United Kingdom; 8 National Institute for Public Health and the Environment (RIVM), Bilthoven, The Netherlands; 9 Department of Gastroenterology and Hepatology, University Medical Centre, Utrecht, The Netherlands; 10 Genomic Epidemiology Group, German Cancer Research Center (DKFZ), Heidelberg, Germany; 11 Andalusian School of Public Health, Granada (Spain) and CIBER de Epidemiología y Salud Pública (CIBERESP), Granada, Spain; 12 Institute of Community Medicine, University of Tromsø, Tromsø, Norway; 13 Fundación Vasca de Innovación e Investigación Sanitarias, Sondika, Bizkaia, Spain; 14 Unit of Nutrition, Environment and Cancer, Catalan Institute of Oncology (ICO)-IDIBELL, Barcelona, Spain; 15 Navarre Public Health Institute, Pamplona, Spain; 16 Consortium for Biomedical Research in Epidemiology and Public Health (CIBER Epidemiología y Salud Pública-CIBERESP), Madrid, Spain; 17 International Prevention Research Institute, Lyon, France; 18 Molecular and Nutritional Epidemiology Unit, Cancer Research and Prevention Institute – ISPO, Florence, Italy; 19 Department of Epidemiology, Regional Health Authority, Murcia, Spain; 20 Department of clinical and experimental medicine, Federico ii University, Naples, Italy; 21 Public Health Directorate, Asturias, Spain; 22 Human Genetics Foundation-HuGeF, Turin, Italy; 23 INSERM U1018, Gustave Roussy Institute, Paris South University, Villejuif, France; 24 Department of Hygiene, Epidemiology and Medical Statistics, Medical School, World Health Organization (WHO) Collaborating Center for Food and Nutrition Policies, University of Athens, Goudi, Athens, Greece; 25 Hellenic Health Foundation, Athens, Greece; 26 Department of Epidemiology, Harvard School of Public Health, Massachusetts, Boston, United States of America; 27 Bureau of Epidemiologic Research, Academy of Athens, Athens, Greece; 28 Potsdam-Rehbrücke Department of Epidemiology, German Institute of Human Nutrition (DIfE), Nuthetal, Germany; 29 Administrative Office of the Commission on Genetic Testing Robert Koch-Institute, Berlin, Germany; 30 Cancer Registry and Histopathology Unit, “Civile M. P. Arezzo” Hospital, Ragusa, Italy; 31 Nutritional Epidemiology Unit, Fondazione IRCCS Istituto Nazionale dei Tumori, Milan, Italy; Centro Cardiologico Monzino IRCCS, Italy

## Abstract

The European Prospective Investigation into Cancer and nutrition (EPIC) is a long-term, multi-centric prospective study in Europe investigating the relationships between cancer and nutrition. This study has served as a basis for a number of Genome-Wide Association Studies (GWAS) and other types of genetic analyses. Over a period of 5 years, 52,256 EPIC DNA samples have been extracted using an automated DNA extraction platform. Here we have evaluated the pre-analytical factors affecting DNA yield, including anthropometric, epidemiological and technical factors such as center of subject recruitment, age, gender, body-mass index, disease case or control status, tobacco consumption, number of aliquots of buffy coat used for DNA extraction, extraction machine or procedure, DNA quantification method, degree of haemolysis and variations in the timing of sample processing. We show that the largest significant variations in DNA yield were observed with degree of haemolysis and with center of subject recruitment. Age, gender, body-mass index, cancer case or control status and tobacco consumption also significantly impacted DNA yield. Feedback from laboratories which have analyzed DNA with different SNP genotyping technologies demonstrate that the vast majority of samples (approximately 88%) performed adequately in different types of assays. To our knowledge this study is the largest to date to evaluate the sources of pre-analytical variations in DNA extracted from peripheral leucocytes. The results provide a strong evidence-based rationale for standardized recommendations on blood collection and processing protocols for large-scale genetic studies.

## Introduction

In recent years, the use of automated methods for DNA extraction from venous blood samples has generated large amounts of material for the mapping of genetic variations that underlie susceptibility to common human diseases [1], [2]. DNA is an abundant molecule in blood (20–60 µg/ml) and is extremely stable after purification. Since most genome-wide analysis methods require ≤1 µg of DNA and for a single SNP assay ≤10ng of DNA, this molecule is rarely in short supply when using blood samples obtained through conventional venipuncture [3]. However in long-term epidemiological studies such as cohort studies, it is essential to maximize the yield and quality of DNA in order to maintain a DNA resource that will last for future research extending over many years. Thus far, there have been only few studies addressing pre-analytical variations affecting the yield of DNA extracted from peripheral blood leucocytes [4].

The European Prospective Investigation into Cancer (EPIC) is a long-term, multi-centric prospective cohort study with a focus on nutrition, investigating the etiology of cancers at various sites as well as other forms of chronic diseases in relation to diet and lifestyle [5]. The study takes advantage of the contrast in cancer rates and dietary habits between centers and countries and of its large overall size, which makes it possible to explore interactions between nutritional, genetic, hormonal and lifestyle factors [6], [7]. The prospective cohort approach includes the collection of baseline questionnaire and interview data on dietary and non-dietary variables, as well as anthropometric measurements and blood samples for long-term storage from apparently healthy populations. The enrollment of subjects in all EPIC centers took place between 1992 and 2000. The cohort participants are followed up over time for the occurrence of cancer and other diseases, as well as for overall mortality, to allow incidence and mortality comparisons by exposure variables. At regular intervals, follow-up questionnaires are used to update information on selected aspects of lifestyle that are known or strongly suspected to be related to cancer risk. To date, EPIC has recruited 521,448 participants, in 23 centers located in 10 European countries. The study started in 1992 with 17 research centers in seven core EPIC countries (France, Germany, Greece, Italy, The Netherlands, Spain and the UK). Subsequently, these were joined by centers in three Scandinavian countries (Sweden, Denmark and Norway) and one center in Italy (Naples) that were conducting broadly similar prospective studies. The most recent follow-up period for cancer incidence was performed between 2004 and 2010, and has identified 50,336 subjects who developed cancer after cohort enrollment (incident cases). These cancers cover a very wide range of anatomic sites and morphologies.

Of the total number of cohort participants, 388,527 have provided a venous blood sample (30 ml) obtained according to standard protocols, which was fractionated into plasma, white blood cells (buffy coat), serum and red blood cells. Except for samples collected in Sweden and Denmark (which were stored locally), aliquots corresponding to 15 ml of fractionated blood were snap-frozen and shipped to a central biobank hosted by the International Agency for Research on Cancer (IARC, Lyon, France). These samples are cryopreserved into liquid nitrogen (−196°C) in plastic straws (Cryobiosystem®).

Here, we have assessed the yield of DNA extracted from approximately 50,000 individual samples collected from individuals in the European Prospective Investigation into Cancer over a period of 5 years, and we have examined the impact of a range of pre-analytical variables on the amount of DNA generated using an automated DNA extraction system.

## Materials and Methods

### Ethics Statement

The DNA extraction data used in the present study relates to projects that have been formally endorsed by the EPIC Steering Committee and approved by the Ethical Review boards of each participating center and of the International Agency for Research on Cancer.

### Subjects

EPIC was constructed by the integration of different cohorts into a common framework. In the majority of study centers, subjects were invited from the general adult population residing in a given town or geographical area. Exceptions to this recruitment scheme were the French cohort (based on members of the health insurance for teacher’s education system), parts of the Italian and Spanish cohorts (based on members of blood donor associations) and the cohorts in Utrecht (The Netherlands) and Florence (Italy) (women invited for a population-based breast cancer screening program). In Oxford (UK) half of the cohort was recruited among vegans (who consume no animal products), lacto-ovo vegetarians and fish eaters (i.e. consumers of fish but not meat). In France, Norway, Utrecht (The Netherlands) and Naples (Italy) only women were recruited. Individuals who agreed to participate signed an informed consent, were mailed a questionnaire on diet and a questionnaire on lifestyle and were subsequently invited to a study center for blood donation, anthropometry and measurement of blood pressure. There were, however, deviations from this general scheme in several centers according to the nature of cohort [5], [8].

### Blood Samples

Thirty ml of blood was obtained by venipuncture and processed according to standard separation protocols. Biological samples included blood plasma, blood serum, white blood cells (buffy coat) and red blood cells were collected from 388,527 of the 521,448****EPIC study participants. In the seven initial EPIC countries and in Naples (Italy), blood fractions were aliquoted into 28 plastic straws containing 0.5ml each (twelve plasma with sodium citrate, eight serum, four erythrocyte, four buffy coat for DNA). Plastic straws (Cryobiosystem® (CBS), Paris, France), made of chemically inert and biocompatible ionomeric resin, were designed for long-term storage. To ensure a high degree of standardization, the same materials (syringes, straws, etc.) were purchased centrally and distributed to the centers. The samples were then split into two mirror halves of 14 aliquots each. One set was stored locally, and one transported to IARC to be stored in liquid nitrogen (at −196°C) in the central biobank.

### Separation of Blood Fractions

The 30 ml of blood collected from each participant were centrifuged while still in the Monovette tube. Centrifugation speed was set at a value that corresponds to a 1500×*g* centrifugal force. During centrifugation for at least 20 minutes the samples were kept at room temperature (±20°C). From the Monovette tubes with anticoagulant, three blood fractions were obtained: plasma, buffy coat, and red blood cells. Buffy coats (2 ml) were adjusted to a final volume of 2.5 ml by addition of physiological solution. From the Monovette without anticoagulant, serum was obtained. After centrifugation of the three Monovette tubes, four plastic tubes were prepared, containing: (1) - 4.5 ml serum, (2) - 6.5 ml plasma, (3) - 2 ml red blood cells +0.5 ml of physiological solution, (4) - 2 ml buffy coat +0.5 ml of physiological solution. Each plastic tube was split into plastic straws containing 500 µl of biological material.

### Sample Storage

The central EPIC biobank located at IARC holds 33 Liquid Nitrogen (LN_2_) tanks equipped with straw storage systems and connected to an automated LN_2_ supply system. The samples are kept under N_2_ liquid phase (−196°C). The biobank contains about 3.8 millions straws with blood aliquots from 275,861 EPIC participants. The straws of each participant are stored together using the CBS™ visotube/goblet/canister system (Cryobiosystem®). Each straw is labeled with the participant’s ID and color-coded to indicate its contents; in addition, the tube, goblet and canister are color-coded to aid in identifying the samples. Finally, a computer software program indicates the container, canister, goblet, and the location of the goblet and the canister within each container to track the stored biological samples of each participant. A Laboratory Information Management System (LIMS) has been used to identify, track and follow-up during analysis the different straws contained in each visotube. The biobank is housed in three purpose-built, ventilated storage rooms. The pressure in the LN_2_ tanks is monitored with alarms. The storage rooms are equipped with LN_2_ sensors to monitor potential LN_2_ health hazards.

### DNA Extraction

Genomic DNA from participants was extracted from one or two aliquots of 0.5 ml aliquot of buffy coat, which had been kept frozen since blood collection and processing. All DNAs were extracted at IARC, Lyon, using the Gentra Autopure LS DNA preparation platform (Qiagen, Hilden, Germany). The two different automated extractors and the manual technique were used applying the same DNA extraction protocol. This purification protocol included 5 steps:

#### RBC lysis

There was an incubation of the sample with 15–19 ml Autopure RBC Lysis solution during 5 min at room temperature to lyse the red blood cells. The samples were then centrifuged at 3000×*g* for 2 min to pellet the white blood cells.

#### Cell lysis and protein precipitation

To disperse the white blood cell pellet, 1.67 ml Autopure Precipitation Solution were vigorously dispensed and then 5 ml Autopure Cell Lysis Solution were added to lyse the white blood cells. The samples were mixed vigorously to precipitate the proteins and then centrifuged at 3000×*g* for 2 min. Five milliliters of Autopure 100% Isopropanol were added to the DNA-containing solution.

#### DNA precipitation

The output tubes were gently rotated 50 times to precipitate the DNA and then the samples were centrifuged at 3000×*g* for 2 min to pellet the DNA.

#### DNA wash

A dispense of 5 ml Autopure 70% Ethanol was done followed by a centrifugation of the samples at 3000×*g* for 1 min to pellet DNA.

#### DNA hydration

DNA was rehydrated with DNA Hydration Solution according the required DNA concentration defined by the users.

### DNA Quantification

Two different methods of quantification were used for measurement of DNA quantity: PicoGreen dsDNA quantitation assay and NanoDrop ND-8000 8 sample spectrophotometer. The PicoGreen dsDNA Quantitation Reagent is an ultra-sensitive fluorescent nucleic acid stain for quantitating double-stranded DNA (dsDNA). DNA samples were pipetted to 96-well plates for DNA concentration measurement with PicoGreen dsDNA quantitation assay and kit (Molecular Probes, Inc, The Netherlands). The NanoDrop ND-8000 8 sample spectrophotometer is a full-spectrum (220–750 nm) instrument that measures 8 individual 1 µl samples.

### Statistical Method

To examine those factors that may be related to DNA yield levels, we modeled DNA yield levels as a linear function of covariates (generalized linear model with gamma distributed outcomes and identity link function). For each variable, results were expressed as regression coefficients reflecting either the increase (positive value) or the decrease (negative value) in DNA yield in relation with the variable under consideration. For categorical variables, coefficients represented the amount of change in DNA yield in µg as compared to the reference category. Age, Body Mass Index (BMI) and processing times were treated as continuous variables. In this case, coefficients represented the amount of change in DNA yield in µg for one unit change (e.g. with each year for age, each BMI unit for BMI, and each 30 minutes for processing times). Analyses were adjusted for the following variables: age, center, gender, BMI, tobacco consumption, number of straws, extraction method and quantification method. Partial R2 was calculated as the sum of squares of an independent variable given other independent variables in the model divided by the residual sum of squares of the model excluding that independent variable and then multiplying by 100 to get a percentage. Analyses were performed using Stata 11.

## Results

### Study Design

This study has used data on DNA extraction processed at IARC and generated in the course of 12 distinct projects developed between 2006 and 2010 using samples of the EPIC cohort (Table 1). Of these projects, 10 were focused on specific cancer cases or etiological risk factors. The two other projects were focused on diabetes (INTERACT) and on heart diseases (EPIC-HEART), respectively. The design of each project was a nested case control study in which ascertained incident cases of disease were selected and matched with controls free of the disease of interest. INTERACT and EPIC-HEART are case-cohort studies, using incident type 2 diabetes for INTERACT, incident coronary heart disease and stroke cases for EPIC-HEART, and a joint referent group which is a random sample of the participants providing blood samples at baseline.

**Table 1:** DNA extraction generated in the course of 12 distinct projects using specimens of the EPIC cohort.

Study code	Study name	Objectives	Numberof DNAextractions
BLAD	Participation in GWAS for bladder cancer	Nested case control study aimed at identifying novel genetic variants which are worthy of intensive pursuit in epidemiological, genetic mapping, clinical and laboratory investigationson bladder cancer.	950
BRCD	Participation in the Breast an Prostate Cancer Cohort Consortium (BPC3) – Breast cancer component	Nested case-control study aimed at the analysis of genes related to steroid hormone and insulin-like growth factor-1 metabolism and breast cancer risk in EPIC which is part of theNCI breast and prostate cancer cohort consortium and GWAS study of ER-negativebreast cancer.	8071
CORD	The Influence of Vitamin D andPolymorphisms of the Vitamin D Receptorand Calcium Sensing Receptoron Colorectal Cancer Risk	Nested case-control study aimed at evaluating the roles of both vitamin-D (important in calcium homeostasis/cell cycle kinetics) and calcium (role in cell cycle kinetics) incolorectal cancer prevention.	2177
EGAD	Genetic susceptibility, environmental factors and the gastric cancer risk in European populations (EUR-GAST II)	Nested case-control study aimed at (a) evaluating the effect of dietary and environmental exposures by histological and anatomical subtypes of gastric cancer; (b) evaluating theeffect of dietary and environmental factors on esophageal adenocarcinomas; (c) evaluatingthe main effect of genetic polymorphisms in several candidates genes.	1444
EPHD	Study of the interplay of genetic, biochemical and lifestyle factors in coronary heart disease (EPIC-HEART)	Nested case-control study aimed at investigating the separate and combined influencesof genetic, biochemical and major lifestyle factors (notably diet) on the incidence ofcoronary heart disease (CHD).	7643
HPVD	HPV and cervical: the role of diet,environmental and infectiouscofactors, and genetic susceptibility	Nested case-control study aimed at evaluating the association between serological markersof HPV infection and cervical cancer as well as the role in cervical carcinogenesis of: (a) environmental cofactors (diet, tobacco, parity, use of hormonal contraceptives),(b) infectious cofactors (HSV-2 and *C. trachomatis)*; and (c) markers of geneticsusceptibility.	664
INTD	Examination of the interaction of genetic and lifestyle factors on the incidence of type 2 diabetes (INTERACT)	Nested case-control study aimed at evaluating gene-lifestyle interactions in relationwith type 2 diabetes.	18439
KIDD	Genome Wide Association Study of kidney cancer	The aims of this study are to (i) immediately replicate approximately the top 30 variants in a large follow-up series, and (ii) substantially replicate between 20,000 and 317,000 variants following the GWAS of kidney cancer recently completed involving 1400 cases and 2800 controls from an IARC Central Europe study.	792
LUND	DNA methylation changes associated with cancer risk factors and blood levels ofvitamin metabolites	The aim of this study is to investigate the contribution of common human genetic variationto susceptibility of lung cancer. The association between lung cancer and DNA methylation patterns in a panel of candidate genes is examined. It is also investigated whether bloodlevels of vitamin metabolites modify DNA methylation levels in blood cells. DNAmethylation levels are quantitatively determined in blood cells of nestedcases and controls.	2450
LYMD	EPIC Nested case-control investigation on lymphomas	Nested case-control study aimed at elucidating whether risk factors for lymphoma exerttheir effect by modulation of the immune system by studying the inherited andacquired immune response in non-Hodgkin lymphoma (NHL) cases and controls.	1789
PAND	Genome Wide association Study andpancreatic cancer (PanScan)	Nested case-control study aimed at conducting a whole genome scan (WGS) of common genetic variants to identify genetic markers of susceptibility to pancreatic cancer.	504
PROD	Participation in the Breast an Prostate Cancer Cohort Consortium (BPC3) – Prostate cancer component	Nested case-control study aimed at the analysis of genes related to steroid hormone and insulin-like growth factor-1 metabolism and prostate cancer risk in EPIC which is part ofthe NCI breast and prostate cancer cohort consortium and GWAS study of aggressiveprostate cancer.	2238

In each project, samples of buffy coat from cases and controls were used for DNA extraction using an automated Autopure LS DNA extraction system (Qiagen, Hilden, Germany). Within the EPIC study, the samples were from participants recruited in 19 different centres (Table S1). Samples from subjects recruited in centres from Denmark and Sweden, (which were not stored or extracted at IARC) were not included in the study.

Data for a total of 52,256 DNA extractions were retrieved in the laboratory database of the IARC Biological Resource Center (BRC) and analyzed for variations with respect to a number of technical, epidemiological or anthropometric factors including center of subject recruitment, age, gender, body-mass index, cancer case or control status, tobacco consumption, number of straws containing buffy coat used for DNA extraction, extraction machine or procedure (two different Autopure instruments were used and a small proportion of the samples were extracted manually), method for DNA quantification (Nanodrop or Picogreen), degree of haemolysis of the blood sample, and variations in the timing of pre-analytical sample processing (time between blood collection by venipuncture and refrigeration at 4°C, time from refrigeration to centrifugation, time from centrifugation to cryopreservation at −80°C). In this study we defined cases as subjects who developed a cancer before or after recruitment, distinguishing between incident cases, corresponding to subjects who developed a cancer during the follow-up period, and prevalent cases, who developed cancer before the recruitment. The major cancer sites were prostate, breast, lung, bladder, colon, kidney, cervix and pancreas. Samples with a DNA yield of 0 were excluded from the statistical analysis (n = 1962). Overall, a total of 47,161 samples were taken into consideration in the final analysis (Table 2).

**Table 2:** Technical, epidemiological and anthropometric factors analyzed for evaluation of DNA yield variations.

Variables		N	%
Gender	Men	18680	39.6
	Women	28481	60.4
Age	<45	7054	15.0
	45–49	6631	14.1
	50–54	8835	18.7
	55–59	9514	20.1
	60–64	8567	18.2
	≥65	6560	13.9
Body Mass Index	Normal (<25)	14449	30.7
	Moderate pre-obesity (25–27.5)	12784	27.1
	Overweight (27.5–30)	7185	15.2
	Moderate obesity (30–35)	8953	19.0
	Obesity (≥35)	2934	6.2
	Missing	856	1.8
Cancer	Incident^a^	10954	23.2
	Non Incident	36207	76.8
	Prevalent^b^	1311	2.8
	Non Prevalent	45850	97.2
Time from blood collection to incident cancer diagnosis	<2 years	1813	3.8
	2–5 years	3299	7.0
	5–10 years	4460	9.5
	≥10 years	1081	2.3
	Missing	36508	77.4
Time from prevalent cancer diagnosis to blood collection	<2 years	240	0.5
	2–5 years	298	0.6
	5–10 years	340	0.7
	≥10 years	427	0.9
	Missing	45856	97.3
	Never	21290	45.1
Tobacco consumption	Former	13847	29.4
	Current	11067	23.5
	Missing	957	2.0
Number of straws	1	11838	25.1
	2	35323	74.9
Extraction method	Extractor LS1	29441	62.4
	Extractor LS2	17305	36.7
	Manual	415	0.9
Quantification method	Nanodrop	33805	71.7
	Picogreen	13356	28.3
Haemolysis	Yes	3337	7.1
	No	21379	45.3
	Missing	22445	47.6
Haemolysis gradient	Light haemolysis	2870	6.08
	Medium haemolysis	445	0.94
	Heavy haemolysis	20	0.05
	Missing	43826	92.93
Time from collection to refrigeration	<5 min	2704	5.7
	5 min - 1 hour	5421	11.5
	1–3 hours	3934	8.3
	>3 hours	3987	8.5
	Missing	31115	66.0
Time from refrigeration to centrifugation	<1.5 hours	498	1.0
	1.5–2 hours	3482	7.4
	2–6 hours	2153	4.6
	≥6 hours	1865	4.0
	Missing	39163	83.0
Time from centrifugation to freezing	<45 min	6179	13.1
	45–59 min	6296	13.4
	1–2 hours	6800	14.4
	≥2 hours	7598	16.1
	Missing	20288	43.0

a First incident cancer case.

b Last prevalent cancer case.

### Sources of Variations in DNA Yield

The average yield of DNA per extraction, given as total amount of DNA recovered after extraction, was 68.85 µg whatever number of straw used (minimum: 1 µg; maximum: 897 µg). When considering DNA extraction from only 1 straw (0.5 ml of buffy coat), the average yield of DNA was 43.23 µg compared to 77.43 µg using 2 straws (Table S2). Table 3 shows the statistical analysis of the effect of anthropometric, epidemiological and technical factors on DNA yield per sample. For each factor, the analysis was adjusted for main variables listed in Table 3 (age, gender, BMI, tobacco consumption, number of straws, extraction and quantification methods) and for center of blood collection.

**Table 3:** Effects of individual characteristics and processing variations on DNA yield (µg).

	Estimated coefficientfor effect^(c)^	SE	P value
**Gender**			
Men	reference		
Women	1.437	0.388	<0.01
**Age**	−0.107	0.020	<0.01
**BMI**	0.390	0.039	<0.01
**Tobacco consumption**			
Never smoker	reference		
Former smoker	0.366	0.375	0.33
Current smoker	10.871	0.515	<0.01
**Incident cancer**			
No	reference		
Yes	2.494	0.363	<0.01
**Previous cancer**			
No	reference		
Yes	1.252	1.157	0.28
**Number of straws used**			
One straw	reference		
Two straws	30.276	0.489	<0.01
**Extraction method**			
Autopure LS 1	reference		
Autopure LS 2	−2.439	0.434	<0.01
Manual	−6.757	0.994	<0.01
**Quantification method**			
Nanodrop	reference		
Picogreen	6.449	0.516	<0.01
**Haemolysis**			
No	reference		
Yes	−7.895	0.814	<0.01
**Haemolysis**			
Light haemolysis	reference		
Medium haemolysis	−5.370	1.685	<0.01
Heavy haemolysis	−9.509	9.221	0.30
**Time from collection to refrigeration** (per 30 minutes)	0.482	0.213	0.02
**Time from refrigeration to centrifugation** (per 30 minutes)	0.227	0.036	<0.01
**Time from centrifugation to freezing** (per 30 minutes)	0.057	0.138	0.68
**Time from blood collection to incident cancer diagnosis** (per year)	−0.138	0.116	0.24
**Time from prevalent cancer diagnosis to blood collection** (per year)	0.358	0.165	0.03

c The estimated coefficients for effect reflect the increasing (positive value) or decreasing (negative value) concentration response to the lifestyle/exposure factor, adjusted for the other lifestyle/exposure factors.

DNA yield was significantly associated with the following individual variables: gender (small but significant increase of 1.44 µg in DNA yield in women), age (overall DNA yield significantly decreased by about 0.11 µg with each year of age), BMI (increase of 0.39 µg in DNA yield with each BMI unit), incident cancer (small but significant increase of 2.49 µg in DNA yield in subjects who developed a cancer during EPIC follow-up) and tobacco consumption (DNA yield significantly increased by 10.87 µg in smokers versus never smokers, non-significant increase in former smokers). The association with incident cancer was not attributable to any specific cancer type or location. In contrast, cancer diagnosis prior to inclusion in EPIC was not significantly associated with changes in DNA yield. Among technical variables, significant changes were observed according to the number of straws used (on average, extraction with 2 straws generated 30.28 µg of DNA more than with 1 straw), center (see below), extraction method (there was a small but significant difference between the two Autopure LS instruments used, and manual extraction had a significantly lower yield than either machine). Detection with Picogreen tended to give higher values than with Nanodrop. This difference appeared to affect DNA yield mostly for extractions performed from one straw of buffy coat. When using 2 straws for extractions, the values obtained with both quantitation methods were similar (75.41 µg with Picogreen versus 77.65 µg with Nanodrop). Processing times were also a significant source of changes. Each 30 minutes of decrease in lag time between blood taking and refrigeration, and between refrigeration and centrifugation, resulted in a significant increase in DNA yield of about 0.23–0.48 µg per sample. In contrast, the time lag between centrifugation and freezing did not appear to have a significant impact on the final DNA yield. It should be noted, however, that about 72% of samples were frozen within a maximum time of 2 hours after centrifugation.

From a technical viewpoint, the main factor negatively affecting DNA yield was haemolysis. The lysis of red blood cells was visually recorded and scored as either “light”, “medium”, or “heavy”. Presence of haemolysis at any degree was associated with a reduction of about 8 µg in DNA yield, with an increasing trend according to the degree of haemolysis. It should be noted, however, that information on haemolysis was recorded for only 24,716 (52.5%) of the samples.

### Variations with Center

**Figure 1:**
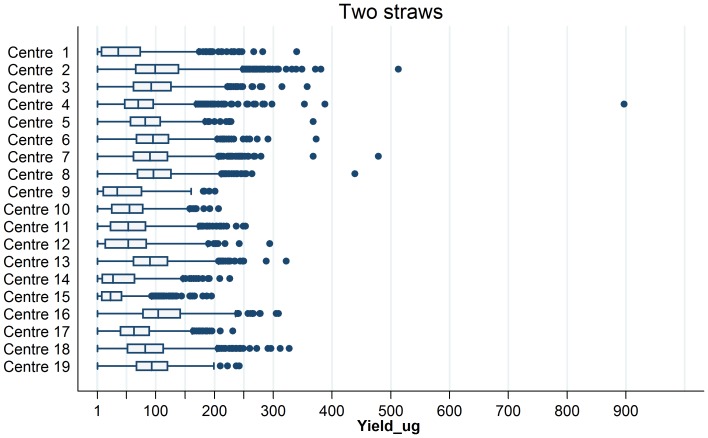
Distribution of yield (µg) for DNA samples extracted with 2 aliquots of buffy coat. Representation of DNA yield for DNA extractions performed from 2 buffy coat aliquots. Boxes extend from 25^th^ to 75^th^ percentiles and are divided by a solid line representing the median of each center. Whiskers extend from lower to upper adjacent values as defined by Tukey. Outliers are denoted by a dot.

Figure 1 shows the variations in the average DNA yield per sample using 2 straws of buffy coat according to the EPIC centre of origin of the sample (for variations in samples extracted using a single straw, see also Table S2). The extent of variation from one center to the other appeared to be as much as fourfold. The mean values of lowest yields (for 2 straws) were detected for center 15 (29.69 µg) and center 14 (40.62 µg) whereas the mean values of highest yields were for center 16 (112.26 µg) and center 2 (104.27 µg) (Table S2). Table S3 shows the statistical analysis of the effect of centre of origin on DNA yield. In several of the centers, there were substantial seasonal variations in DNA yield according to the date when the blood sample was collected, in particular in those with the lowest yield (Figure S1). These seasonal intra-center variations, independently of other processing variables, suggest that in some centers there were significant differences in the separation, recovery and aliquoting of buffy coats. Partial R^2^ analysis identified that the variable “Center” accounted for 16.9% (P<0.0001) of the explained variance, making it the most important significant predictor for DNA yield.

### Qualification for Genotyping Studies

The DNA extracted from EPIC samples has been used in Genome-Wide Association Studies (GWAS) and candidate gene studies by different laboratories (Table 1). The methodology used by laboratories performing GWAS was based either on Illumina or Sequenom technologies using different types of SNP arrays. Each laboratory developed its own quality controls procedures, depending upon the genotyping methodology used. Tables 4 and 5 compile information on samples used in four studies completed to date and show the percentage of those samples which met qualification criteria as determined by the different laboratories to generate exploitable SNP data (DNA amount, concentration). For these analyses, samples with DNA yield  = 0 were retained. Different laboratories have used different criteria depending on their particular technology setups. Notably the amount of DNA required for qualification was different among studies. Table 4 shows that three studies required between 50 ng and 1.25 µg of DNA to perform genotyping analyses (KIDD (qualification 100%), PAND (96.32%) and BRCD/PROD (100%)) whereas INTD required samples with more than 10 µg of DNA at a concentration ≥10 ng/µl, thus explaining the lower qualification rate (84.02%) due to samples with low concentration/yield. Aside from amount of DNA, other reasons for non-qualification were gender discordance between sample annotation and quality control assessment (between 0.42 and 0.97%) and low SNP call rate (between 0.53 and 2.76%), depending upon studies (Table 5). Thus, the main reason for non-qualification was insufficient DNA yield and concentration.

**Table 4:** Inclusion criteria for genotyping projects.

		Criteria		Number of samples excluded			
Project	Number of samples	Quantity	Concentration	Insufficientyield	Lowconcentration	% of samples failed	% of samples qualified for genotyping
Kidney (KIDD)	258	50 ng	50 ng/µl	0	0	0	100
PanScan (PAND)	489	1250 ng	25 ng/ul	n/a	18	3.68	96.32
BPC3 (BRCD+PROD)	5684	250 ng	50 ng/µl	0	0	0	100
Interact (INTD)^d^	20794	10 µg	50 ng/ul	433	2889	15.98	84.02


d Samples not having the required amount of DNA (with less than 10 µg of DNA) were sent to the laboratory.

**Table 5:** Qualification for different genotyping method.

Project	Genotypingmethod	Platform/technology	Site	Number ofsamples selectedfor genotyping	% of samplesgenotypedthat passed	Failedgenotyping	Criteria
Kidney (KIDD)	GWAS	Illumina Infinium 610 K	CNG, Evry, France	258	100.00	0	
PanScan(PAND)	GWAS	Illumina Infinium IIHuman 550 K Bead	NCI, Bethesda,USA	471	96.82	15	<98% call rate(n = 13, 2.76%),gender (n = 2, 0.42%)
BPC3(BRCD+PROD)	GWAS	Illumina Golden Gate	ICL, London, UK	5684	99.47	30	<75% call rate(n = 30, 0.53%)
Interact (INTD)	I-plex	Sequenom	MRC, Cambridge,UK	17472	98.48	265	<75% call rate(n = 96, 0.55%),gender (n = 169, 0.97%)

## Discussion

This study is, to our knowledge, the largest to date to evaluate the sources of pre-analytical variations in DNA extracted from blood samples for Genome Wide Analysis Studies. So far, there has been no systematic assessment of these sources of variations. Early studies discussing these variations have merely listed steps in the procedure and factors that may affect DNA yield without quantifying their respective impact [9]. In contrast, recent studies have evaluated the use for GWAS of DNA of different source/quality to compare their suitability, without discussing the impact of sources of variations [10]. Our study is unique in its relatively homogenous study design and infrastructure context (the EPIC cohort and the IARC DNA extraction facility), in which it is expected that sources of variations would be relatively well controlled. In particular, the EPIC centers included in this study have adopted standardized protocols for blood collection by venipuncture, processing, aliquoting and shipment to IARC. Furthermore, DNA extractions at IARC were processed in a single pipe-line with the majority being processed using automated DNA extraction technology.

The large numbers of samples compiled in this study has allowed us to identify several factors that significantly impact on the DNA yield. Importantly, the largest variation in DNA yield was observed between centers, accounting up to 16.9% of the explained variation in DNA yield. The reasons for these inter-center variations may reside in multiple components of the laboratory setting, including room temperature, transport conditions between the place of blood taking and the processing laboratory, the performance of the centrifuge and, importantly, the skills of the laboratory staff to identify buffy coats and effectively recover them in an adequate manner. Although considered as a simple procedure, buffy coats are difficult to identify and recover manually in a reproducible way. The buffy coat interface can be fuzzy and sometimes barely visible. Their size and distribution in the centrifuge tube may also be affected by blood viscosity. Furthermore, the buffy coat layer is unstable and might be perturbed by brisk manipulation of the tube after centrifugation.

After adjustment for main variables (center, age, gender, BMI, tobacco consumption, number of straws, extraction method and quantification method), three individual factors had a measurable and significant effect: gender, age and body mass index. The difference in DNA yield between women and men is small (the yield in women is, on average, 1.4 µg higher than in males) and might be related to variation in lymphocyte, platelets and neutrophils counts (all higher in females than in males) [11]. Similarly, the decrease in DNA yields in relation to age might be caused by decreased number of white cells in the peripheral circulation with age. The decrease in yield might also be influenced by differences in the composition of the white blood cells (WBC) pool that may modify the appearance and thus the retrieval, of the buffy coat. Many years ago, Erkeller-Yuksel and collaborators have studied the age-related changes in human blood lymphocytes subpopulations [12]. They showed that the decrease in lymphocytes counts with age is progressive in all 5-years age groups and that there is no significant acceleration in older subjects. Richardson and collaborators reported similar findings in their study on the evaluation of the effects of blood storage at 4°C on the DNA yield and quality [13]. In this study the main determinant on DNA yield was the age of the participant in the study, with older persons having a lower DNA yield.

With BMI, using a BMI of 25 or under as reference, we found a progressive increase in DNA yield independently of age, the largest increment being detected in highly obese subjects with a BMI ≥35 Kg/M^2^. This increase is likely to be due to an increased number of inflammatory, reactive white blood cells in relation to obesity, a phenomenon which is well documented [14].

DNA yield was affected by tobacco consumption. The DNA recovered from buffy coat was increased by 15.8% in smokers compared to non-smokers. An increase in WBC counts has been documented in smokers, especially leucocytes or lymphocytes subpopulations among smokers [15]. Conversely, smoking cessation has been shown to result into decreased WBC counts to levels comparable to those of never smokers [16], [17]. The increase in WBC counts and subsequent DNA yield in smokers might be caused by chronic inflammation induced by tobacco and is consistent with the hypothesis that blood-derived DNA might represent a source of biomarkers of molecular changes associated with smoking.

We also found that DNA yield varied significantly according to the cancer case or control status of the subjects. Incident cancer cases had, on average, a greater yield of DNA. This difference was relatively small as compared to the average DNA yield, precluding the use of increased DNA yield as an indicator of cancer risk at the individual level (about 4%). Nevertheless, this increase was strongly statistically significant even after adjusting for all other factors including those that might predict higher cancer risk (age, BMI, tobacco). This increase in DNA yield in subjects who will develop cancer during follow-up (mean follow-up time: 5.2 years) might be due to the expansion of pools of WBC involved in cancer-specific immune response, and/or to an increase in inflammatory cells; whereas a contribution of circulating cells originating from an undiagnosed, early lesion can also not be excluded. In breast cancer patients, for example, it has been shown that circulating tumor cell (CTC) assessment could be an indicator of disease progression [18]. Interestingly, there was no significant difference in DNA yield for subjects with prevalent cancer (that is, subjects who had a diagnosis of cancer before recruitment into EPIC). This observation suggests that DNA yields return to basal levels in apparently disease-free cancer survivors.

The other sources of DNA yield variations identified in this study are of a technical nature. Some of these variations can be associated with pre-analytical DNA processing. Interestingly, the times elapsed between blood draw and refrigeration and between refrigeration and centrifugation had an impact on DNA yield, albeit these effects were relatively small. It should be considered that, in the EPIC protocol, these time periods were carefully monitored and controlled in order to minimize variation. Larger variations might be expected in studies where sample collections are assembled from centers that do not use protocols agreed upon by all centers at the onset of the study. Another, minor source of variation was observed in relation to the two automated DNA extractors that were used throughout these studies, indicating that they have slightly different performances. The yield with the automated DNA extractors was higher than for the samples extracted manually using Gentra Puregene columns (Qiagen, Hilden, Germany). However the number of DNA samples that were manually extracted represented less than 1% of the total (415/47,161). Manual methods were used only as a backup when automated extractors were undergoing maintenance or repair.

In the present series of samples, most extractions were performed using 2 straws of buffy coat (74.8%) while a minority was performed using a single straw. Strikingly, the amount of DNA obtained from 2 straws was systematically less than double the amount obtained from one straw. The difference between one and two straws was, on average, 79.13%. This apparent inconsistency might be explained by inequality between the two straws in terms of the quantity of buffy coat material. Whereas the standard EPIC protocol included the collection of 4 identical straws of buffy coat (2 to be stored at the collection center and 2 in the central EPIC biobank at IARC), variations in the filling of the straws might have occurred in particular when buffy coats were in short supply. Extraction from a single straw was often performed from the “most filled of two” available straws as judged by eye by the technicians responsible for sample recovery in the liquid nitrogen tanks.

We also detected variations in relation with to the method used for DNA quantification. Overall, Picogreen detection tended to provide higher yields than Nanodrop, an unexpected observation since others have reported that Nanodrop tended to overestimate DNA yields due to insufficient discrimination between double stranded DNA and single stranded nucleic acids [19]. Furthermore, the difference we observed was essentially for samples with low DNA yields, since when using 2 straws for extractions, the values obtained with both quantitation methods were similar (75.4 µg with Picogreen versus 77.6 µg with Nanodrop). Further studies are needed to fully assess the extent of the differences between Picogreen and Nanodrop quantitation over a wide range of DNA concentrations.

The largest sources of variation were the degree of sample haemolysis and the center from which the sample originated. The scoring of haemolysis was based on a simple visual, qualitative assessment. Furthermore, data are missing for about half of the samples. Given that heavy haemolysis appears to cause a decrease in DNA yield of about 13.8%, this factor may be a non-negligible cause of variations in DNA yield and should be taken into account in annotating sample quality in biobank databases.

Genotyping data indicated that the vast majority of samples performed adequately in different types of SNP assays (pass rate between 84.02% and 100%). There are two main reasons for failures, samples failing to be included in genotyping because of DNA quantity and samples excluded from genotyping results due to SNP call rate or gender error. The first reason for failure was insufficient DNA amount or concentration as measured by the laboratory which performed the assay. This factor had a particularly important impact for the INTD study (n = 20.794), being responsible for 15.98% of the failures in this particular study. In this respect, INTD was different from the three other studies for which genotyping data are currently available, since the laboratory performing the genome-wide INTD study required 10 µg of DNA and a concentration ≥10 ng/µl, a much higher level than other studies such as PAND or KIDD which required only up to 1.25 µg of DNA. Moreover for KIDD, PAND and BRCD/PROD, samples not having the required amount of DNA were not sent by the IARC BRC to the laboratory whereas for INTD, all samples including those with less than 10 µg of DNA were sent to the laboratory. This particularity for INTD explains the lower percentage of samples qualified for genotyping (84.02% for INTD compared to 100% for KIDD and BRCD+PROD, and 96.32% for PAND).

Once qualified, only between 0.53% and 2.76% of samples failed the genotyping procedure. The second reason for failure is due to SNP call rate or gender discordance. It is important to note that there is very little information on the extent of inter-laboratory variations in GWAS studies, most studies on the repeatability being focused on statistical considerations for SNP calling. Our results emphasize the fact that other factors, including particular DNA quality and methods for determining which samples qualify for GWAS analysis, may have a significant impact as sources of possible variations.

In summary, this study uses a very large set of DNA extraction data from a single cohort study (EPIC) to identify several anthropometric, epidemiological and technical factors that influence the overall DNA yield using an automated DNA extraction procedure. Although the vast majority of the samples met the qualification criteria for genotyping studies in different laboratory contexts, the results presented here will provide a strong basis for further recommendation in order to improve blood collection and processing protocols in large-scale genetic studies. In particular, it will be essential to develop simple and cheap tests to assess the quality of buffy coat recovery prior to storage and DNA extraction or where possible to use automated methods for extraction of DNA from whole blood. Another option could be to investigate the benefit of using stand alone automated devices for the identification and transfer of buffy coats. Finally, our results highlight the importance of adequate training and quality control procedures for minimizing inter-center variations as well as temporal variations within each center.

## Supporting Information

Figure S1 **Temporal variations in DNA yield in several centers.**
(PDF)

Table S1 **Distribution of buffy coat selected for DNA extraction among the 19 participating EPIC centers.**
(DOC)

Table S2 **Effects of sample origin (center) on DNA yield (µg).**
(DOC)

Table S3 **Quantities of DNA extracted by center according the number of buffy coats aliquots used.**
(DOC)
