# Supplementary figures and images for: Sources of Pre-Analytical Variations in Yield of DNA Extracted from Blood Samples: Analysis of 50,000 DNA Samples in EPIC
Source: PLoS One. 2012 Jul 13;7(7):e39821. doi: 10.1371/journal.pone.0039821 (PMC3396633; doi:10.1371/journal.pone.0039821)

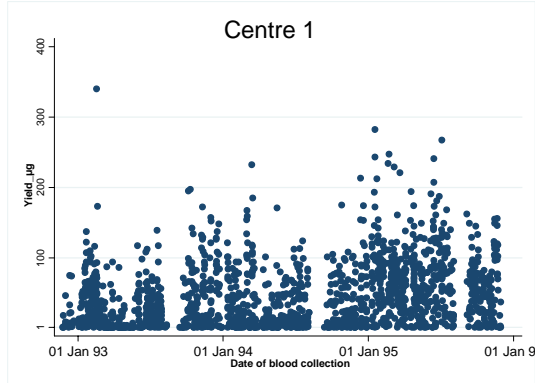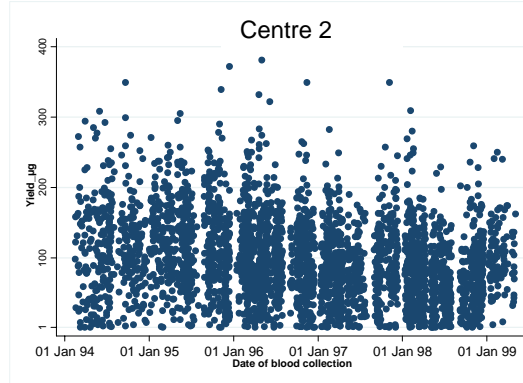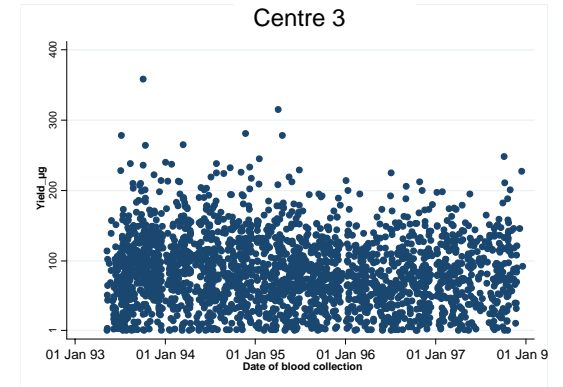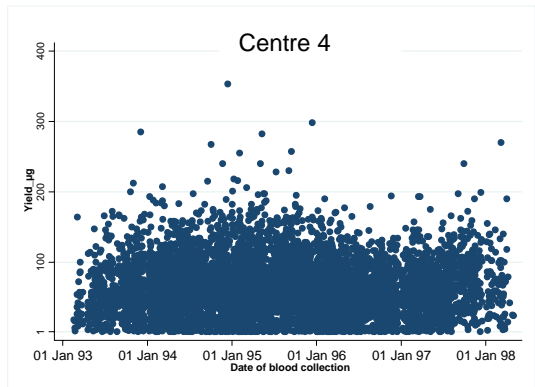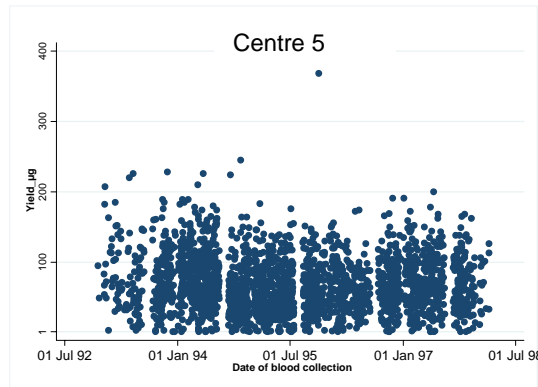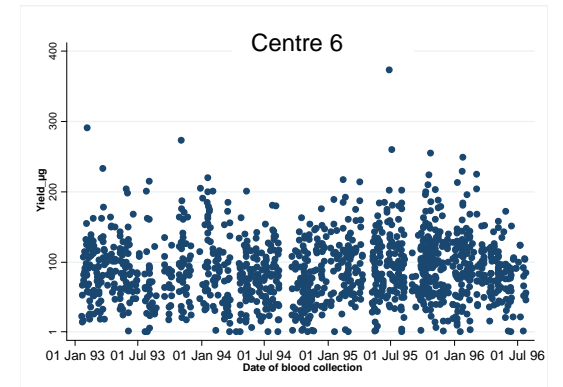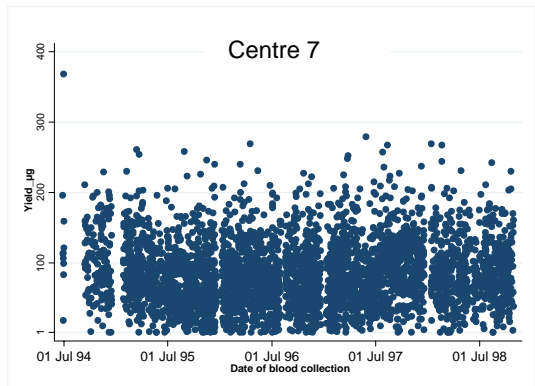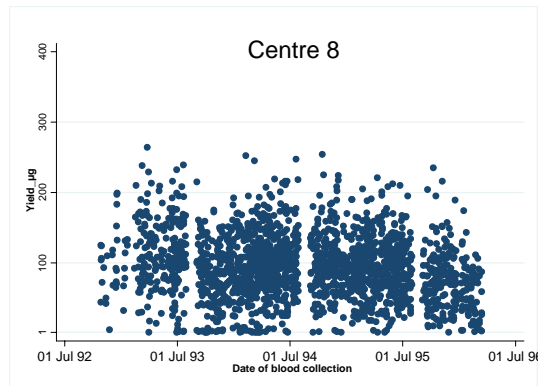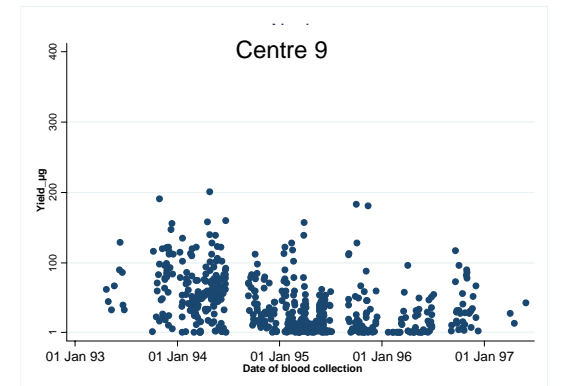

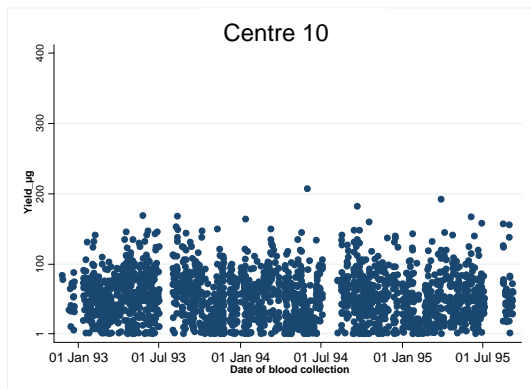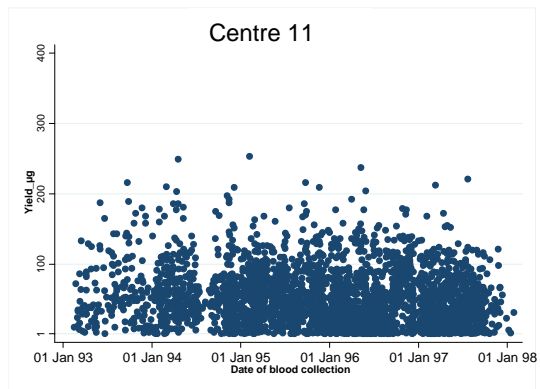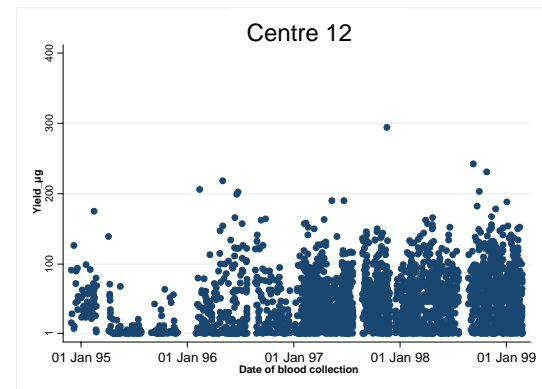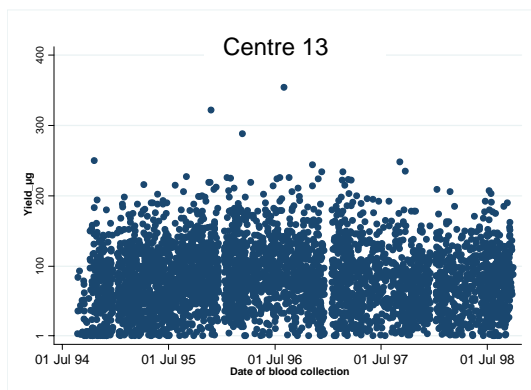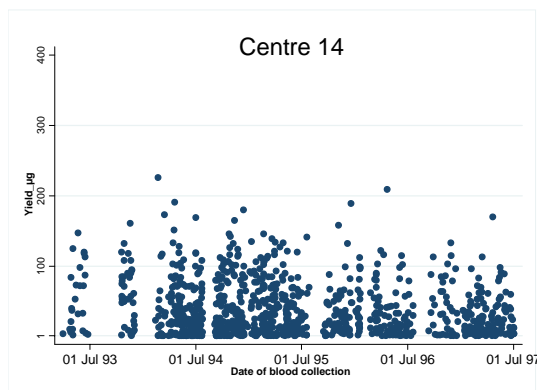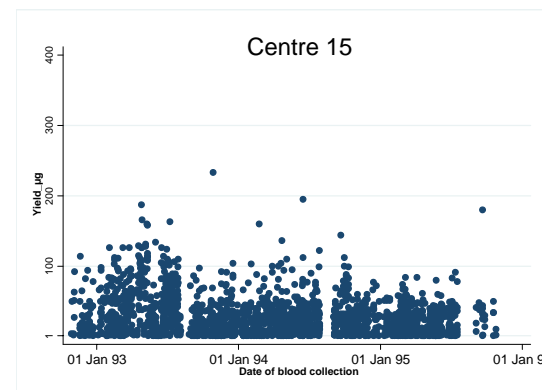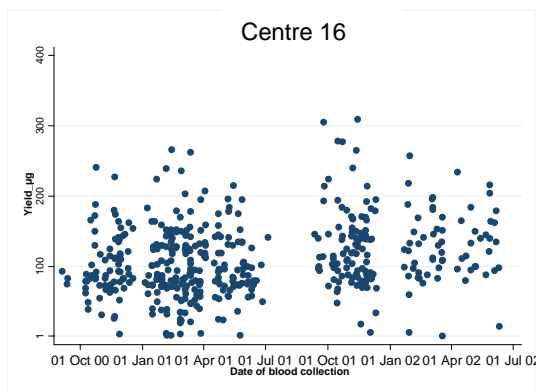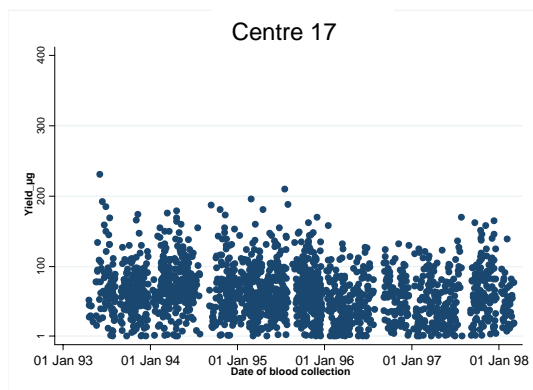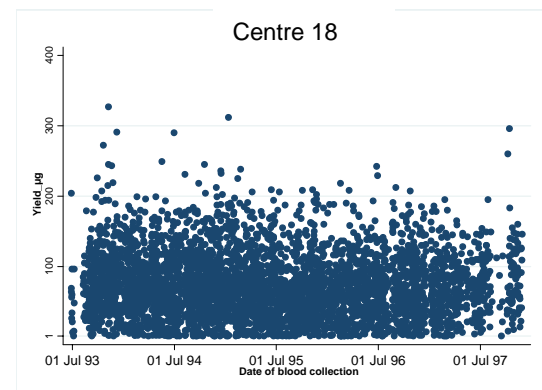

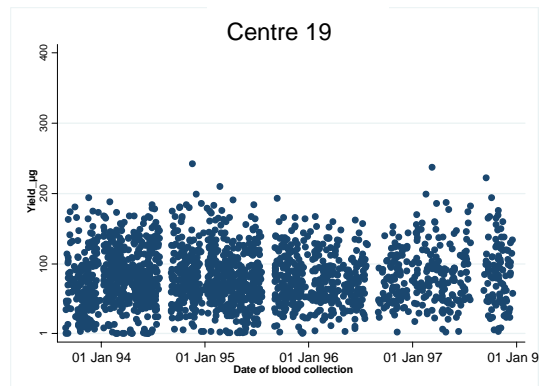

Figure S1: Temporal variations in DNA yield in several centers

Supplement: Figure S1 — Temporal variations in DNA yield in several centers. (PDF) [file pone.0039821.s001.pdf]
