# Supplementary material for: Sources of Pre-Analytical Variations in Yield of DNA Extracted from Blood Samples: Analysis of 50,000 DNA Samples in EPIC
Source: PLoS One. 2012 Jul 13;7(7):e39821. doi: 10.1371/journal.pone.0039821 (PMC3396633; doi:10.1371/journal.pone.0039821)
Supplement: Table S1 — Distribution of buffy coat selected for DNA extraction among the 19 participating EPIC centers. (DOC) [file pone.0039821.s002.doc]

**Table S1**

Distribution of buffy coat selected for DNA extraction among the 19 participating EPIC centers.

| **Country** | **Centers** | **N** | **%** |
| --- | --- | --- | --- |
| France | Paris | 2779 | 5.89 |
| Germany | Heidelberg | 3869 | 8.20 |
|  | Potsdam | 4142 | 8.78 |
| Greece | Athens | 3390 | 7.19 |
| Italy | Florence | 2350 | 4.98 |
|  | Naples | 536 | 1.14 |
|  | Ragusa | 1001 | 2.12 |
|  | Turin | 1882 | 3.99 |
|  | Varese | 2020 | 4.28 |
| Norway | Tromsø | 457 | 0.97 |
| Spain | Asturias | 1654 | 3.51 |
|  | Granada | 1290 | 2.74 |
|  | Murcia | 1900 | 4.03 |
|  | Navarra | 2113 | 4.48 |
|  | San Sebastian | 2093 | 4.44 |
| The Netherlands | Bilthoven | 2331 | 4.94 |
|  | Utrecht | 4152 | 8.80 |
| United Kingdom | Cambridge | 6308 | 13.38 |
|  | Oxford | 2894 | 6.14 |
| **TOTAL** | | **47161** | **100** |
