# Supplementary material for: Sources of Pre-Analytical Variations in Yield of DNA Extracted from Blood Samples: Analysis of 50,000 DNA Samples in EPIC
Source: PLoS One. 2012 Jul 13;7(7):e39821. doi: 10.1371/journal.pone.0039821 (PMC3396633; doi:10.1371/journal.pone.0039821)
Supplement: Table S2 — Effects of sample origin (center) on DNA yield (µg). (DOC) [file pone.0039821.s003.doc]

Table S2

Quantities of DNA extracted by center according the number of buffy coats aliquots used.

| **Centre** | **Number of aliquots** | **Number of samples** | **Mean** | **Mediane** | **25% percentile** | **75% percentile** | **Min** | **Max** | **Standard deviation** |
| --- | --- | --- | --- | --- | --- | --- | --- | --- | --- |
| 1 | 1 | 282 | 27.23 | 16 | 4 | 41 | 1 | 140 | 30.08 |
|  | 2 | 1372 | 47.26 | 36 | 7.5 | 74 | 1 | 340 | 46.48 |
|  | **Average** |  | **43.84** | **30** | **6** | **69** | **1** | **340** | **44.75** |
| 2 | 1 | 391 | 51.17 | 46 | 26 | 72 | 1 | 214 | 31.81 |
|  | 2 | 2999 | 104.27 | 99 | 66 | 139 | 1 | 513 | 57.78 |
|  | **Average** |  | **98.15** | **92** | **57** | **133** | **1** | **513** | **57.95** |
| 3 | 1 | 523 | 47.83 | 46 | 27 | 64 | 1 | 177 | 28.23 |
|  | 2 | 1808 | 94.30 | 92 | 62 | 126 | 1 | 358 | 50.35 |
|  | **Average** |  | **83.84** | **80** | **48** | **116** | **1** | **358** | **50.2** |
| 4 | 1 | 1645 | 36.72 | 35 | 19 | 50 | 1 | 182 | 24.07 |
|  | 2 | 4663 | 73.48 | 70 | 47 | 96 | 1 | 897 | 41.17 |
|  | **Average** |  | **63.90** | **59** | **36** | **88** | **1** | **897** | **40.8** |
| 5 | 1 | 926 | 45.42 | 44 | 28 | 58 | 1 | 245 | 24.98 |
|  | 2 | 1424 | 83.02 | 82 | 57 | 108 | 1 | 368 | 40.47 |
|  | **Average** |  | **68.21** | **63** | **39** | **93** | **1** | **368** | **39.69** |
| 6 | 1 | 240 | 53.52 | 52.5 | 38 | 69 | 1 | 164 | 24.18 |
|  | 2 | 1050 | 96.76 | 95 | 67 | 122 | 1 | 373 | 45.76 |
|  | **Average** |  | **88.71** | **85** | **57** | **116** | **1** | **373** | **45.78** |
| 7 | 1 | 962 | 55.78 | 53 | 36 | 72 | 1 | 261 | 30.98 |
|  | 2 | 2907 | 93.05 | 90 | 62 | 120 | 1 | 479 | 46.22 |
|  | **Average** |  | **83.80** | **79** | **52** | **110** | **1** | **479** | **45.85** |
| 8 | 1 | 206 | 55.02 | 51 | 35 | 73 | 1 | 177 | 28.82 |
|  | 2 | 1694 | 98.28 | 96 | 69 | 126 | 1 | 439 | 46.17 |
|  | **Average** |  | **93.59** | **92** | **62** | **122** | **1** | **439** | **46.59** |
| 9 | 1 | 216 | 29.32 | 24 | 6 | 44.5 | 1 | 121 | 26.8 |
|  | 2 | 320 | 47.18 | 34.5 | 10.5 | 76 | 1 | 201 | 43.18 |
|  | **Average** |  | **39.98** | **29.5** | **8** | **60** | **1** | **201** | **38.44** |
| 10 | 1 | 333 | 35.52 | 35 | 20 | 49 | 1 | 135 | 21.6 |
|  | 2 | 1780 | 54.68 | 55 | 25 | 78 | 1 | 207 | 35.44 |
|  | **Average** |  | **51.66** | **50** | **24** | **74** | **1** | **207** | **34.36** |
| 11 | 1 | 911 | 35.97 | 32 | 16 | 50 | 1 | 730 | 33.73 |
|  | 2 | 1983 | 57.95 | 53 | 23 | 83 | 1 | 253 | 42.65 |
|  | **Average** |  | **51.03** | **44** | **20** | **72** | **1** | **730** | **41.34** |
| 12 | 1 | 1234 | 31.22 | 25.5 | 6 | 49 | 1 | 231 | 28.72 |
|  | 2 | 1545 | 54.81 | 53 | 14 | 84 | 1 | 294 | 42.73 |
|  | **Average** |  | **44.34** | **38** | **10** | **68** | **1** | **294** | **38.97** |
| 13 | 1 | 1017 | 51.65 | 49 | 32 | 68 | 1 | 354 | 30.43 |
|  | 2 | 3125 | 90.91 | 90 | 62 | 120 | 1 | 322 | 45.33 |
|  | **Average** |  | **81.27** | **79** | **48** | **109** | **1** | **354** | **45.42** |
| 14 | 1 | 169 | 21.04 | 12 | 4 | 30 | 1 | 170 | 23.75 |
|  | 2 | 832 | 40.62 | 27 | 9 | 64 | 1 | 226 | 39.22 |
|  | **Average** |  | **37.31** | **24** | **8** | **56** | **1** | **226** | **37.77** |
| 15 | 1 | 348 | 22.32 | 18 | 7 | 30 | 1 | 233 | 22.4 |
|  | 2 | 1745 | 29.69 | 23 | 8 | 42 | 1 | 195 | 28.47 |
|  | **Average** |  | **28.47** | **21** | **7** | **39** | **1** | **233** | **27.69** |
| 16 | 1 | 9 | 68.56 | 52 | 15 | 128 | 1 | 154 | 58.89 |
|  | 2 | 448 | 112.26 | 104 | 78 | 142 | 1 | 309 | 51.62 |
|  | **Average** |  | **111.39** | **103** | **78** | **142** | **1** | **309** | **52.06** |
| 17 | 1 | 272 | 39.03 | 37 | 23 | 53 | 1 | 127 | 21.66 |
|  | 2 | 1610 | 65.90 | 63 | 40 | 169 | 1 | 231 | 36.88 |
|  | **Average** |  | **62.01** | **59** | **36** | **84** | **1** | **231** | **36.34** |
| 18 | 1 | 1403 | 49.41 | 47 | 31 | 65 | 1 | 290 | 27.2 |
|  | 2 | 2749 | 84.07 | 82 | 52 | 113 | 1 | 327 | 47.9 |
|  | **Average** |  | **72.37** | **67** | **39** | **99** | **1** | **327** | **45.13** |
| 19 | 1 | 751 | 59.71 | 58 | 43 | 75 | 1 | 183 | 26.22 |
|  | 2 | 1269 | 93.26 | 93 | 67 | 120 | 1 | 242 | 40.95 |
|  | **Average** |  | **80.79** | **78** | **52** | **106** | **1** | **242** | **39.64** |
| **All centers** | **1** | **11838** | **43.23** | **41** | **21** | **60** | **1** | **730** | **29.58** |
|  | **2** | **35323** | **77.43** | **75** | **41** | **107** | **1** | **897** | **49.19** |
|  | **Average** |  | **68.85** | **63** | **33** | **97** | **1** | **897** | **47.46** |
