# Supplementary material for: Sources of Pre-Analytical Variations in Yield of DNA Extracted from Blood Samples: Analysis of 50,000 DNA Samples in EPIC
Source: PLoS One. 2012 Jul 13;7(7):e39821. doi: 10.1371/journal.pone.0039821 (PMC3396633; doi:10.1371/journal.pone.0039821)
Supplement: Table S3 — Quantities of DNA extracted by center according the number of buffy coats aliquots used. (DOC) [file pone.0039821.s004.doc]

**Table S3**

Effects of sample origin (center) on DNA yield (µg).

| **Center** | **Estimated coefficient for effect (*)** | **SE** | **P value** |
| --- | --- | --- | --- |
| Centre 1 | reference |  |  |
| Centre 2 | 49.39 | 1.25 | <0.01 |
| Centre 3 | 34.61 | 1.23 | <0.01 |
| Centre 4 | 20.12 | 0.81 | <0.01 |
| Centre 5 | 25.39 | 1.04 | <0.01 |
| Centre 6 | 42.77 | 1.67 | <0.01 |
| Centre 7 | 38.38 | 1.04 | <0.01 |
| Centre 8 | 44.73 | 1.50 | <0.01 |
| Centre 9 | 2.83 | 1.13 | <0.01 |
| Centre 10 | 5.87 | 0.90 | <0.01 |
| Centre 11 | 11.37 | 0.82 | <0.01 |
| Centre 12 | 7.18 | 0.81 | <0.01 |
| Centre 13 | 35.81 | 0.99 | <0.01 |
| Centre 14 | 5.08 | 0.84 | <0.01 |
| Centre 15 | -9.04 | 0.68 | <0.01 |
| Centre 16 | 58.72 | 3.43 | <0.01 |
| Centre 17 | 15.54 | 1.06 | <0.01 |
| Centre 18 | 28.76 | 0.95 | <0.01 |
| Centre 19 | 38.43 | 1.27 | <0.01 |

Adjustment for age, gender, BMI, tobacco consumption, number of straws, extraction method and quantification method.
